# A reliable Raman-spectroscopy-based approach for diagnosis, classification and follow-up of B-cell acute lymphoblastic leukemia

**DOI:** 10.1038/srep24821

**Published:** 2016-04-19

**Authors:** Stefano Managò, Carmen Valente, Peppino Mirabelli, Diego Circolo, Filomena Basile, Daniela Corda, Anna Chiara De Luca

**Affiliations:** 1 Institute of Protein Biochemistry, National Research Council, Via P. Castellino 111, 80131 Naples, Italy; 2 IRCCS SDN Istituto di Ricerca Diagnostica e Nucleare, Via E. Gianturco 113, 80143 Naples, Italy; 3 UOSC Servizio di Immunoematologia e Medicina Trasfusionale, AORN Cardarelli Hospital, Via A. Cardarelli 9, 80131, Naples, Italy

## Abstract

Acute lymphoblastic leukemia type B (B-ALL) is a neoplastic disorder that shows high mortality rates due to immature lymphocyte B-cell proliferation. B-ALL diagnosis requires identification and classification of the leukemia cells. Here, we demonstrate the use of Raman spectroscopy to discriminate normal lymphocytic B-cells from three different B-leukemia transformed cell lines (i.e., RS4;11, REH, MN60 cells) based on their biochemical features. In combination with immunofluorescence and Western blotting, we show that these Raman markers reflect the relative changes in the potential biological markers from cell surface antigens, cytoplasmic proteins, and DNA content and correlate with the lymphoblastic B-cell maturation/differentiation stages. Our study demonstrates the potential of this technique for classification of B-leukemia cells into the different differentiation/maturation stages, as well as for the identification of key biochemical changes under chemotherapeutic treatments. Finally, preliminary results from clinical samples indicate high consistency of, and potential applications for, this Raman spectroscopy approach.

Acute lymphoblastic leukemia type B (B-ALL) is a neoplastic disorder that shows the highest childhood cancer-related mortality^1^. It is characterized by immature B-cell progenitors (i.e., lymphoid or lymphoblastic cells) that cannot mature properly into lymphocytic B cells^1^,^2^. B-ALL is a hematological malignancy that is characterized by uncontrolled and rapid cell proliferation. Thus, its timely and accurate diagnosis is fundamental for successful clinical treatment.

A firm diagnosis of B-ALL requires first the identification of the leukemia cells, and second their classification based on the differentiation/maturation stage in which the lymphoblastic B cells are blocked. B-ALL classification is primarily achieved by morphological and immunophenotypic analyses of cell samples from bone marrow or peripheral blood^1^,^2^,^3^,^4^,^5^. Morphological approaches allow the identification of B-ALL lymphoblasts and their classification into three main types: (i) L1 blasts, with small and homogenous cell size, high nuclear/cytoplasmic ratio, and unclear nucleoli; (ii) L2 blasts, with medium cell size, lower nuclear/cytoplasmic ratio, with one or more visible nucleoli; and (iii) L3 blasts, with larger and pleomorphic cell size, prominent nucleoli, and abundant cytoplasm. However, in some cases of poorly differentiated B-ALL, morphological assessment provides low sensitivity and equivocal results^6^. Although most cases can be diagnosed by this method, there is only a modest correlation between morphological categories, treatment responsiveness, and prognosis^6^. Detection of specific antigens that are related to these maturation stages might have prognostic or therapeutic implications, even within a single acute leukemia subtype.

As a consequence, this morphological approach can be combined with immunophenotypic B-ALL cell analysis of the arrested stage of B-cell maturation in terms of the surface expression of up to six to eight different B-cell–associated antigens by multi-parametric flow cytometry^7^,^8^,^9^,^10^. Using this method, the B-ALL cell lineage is currently defined as: (i) ‘pro–B-ALL’, when the cells originate from early pro–B lymphoblasts that express CD19 and CD38 at the plasma membrane; (ii) ‘common B-ALL’, when the cells originate from late pro–B lymphoblasts or intermediate B-cell precursors, as identified by the expression of CD19, CD38, CD10, and CD79a at the plasma membrane; and (iii) ‘pre–B-ALL’, when the cells originate from more committed progenitors defined as pre–B lymphoblasts that express CD19, CD38, CD10, CD79a, CD20, CD22, and immunoglobulins at the plasma membrane^7^. However, this immunophenotypic analysis requires a panel of antibodies against several lymphoid-expressing antigens, and it is labor intensive and time consuming. Moreover, the use of fluorescent dyes is frequently limited by photobleaching of the dye molecule, the limited ability to detect multiple dyes, and interference with the fluorescence of the routine stains used in the cell morphology assessment^11^. Therefore, new approaches are required for rapid and sensitive diagnosis, classification, and prognosis of leukemias.

In the last 10 to 15 years, photonic techniques have emerged as powerful tools for determination of the invasiveness of cancer tissues during surgery^12^ and for the study of the responses of biosystems at the single-cell level^13^. Indeed these methods are non-invasive^14^, and they offer single-molecule detection sensitivity^15^,^16^. This allows functional imaging at micrometer, and even nanometer, resolution^17^,^18^,^19^, while not interfering with existing techniques, thereby increasing the likelihood of their use in a clinical setting.

In terms of a label-free method, Raman spectroscopy (RS) is more attractive than fluorescence because it detects the vibrations of the chemical bonds in molecules through inelastic scattering of light^20^. RS thus provides specific information that is related to nucleic acids, proteins, carbohydrates, and lipids within the cell^21^, and it does not require any external labeling^22^. A typical Raman spectrum functions as a molecular fingerprint of a cell, by providing chemical information that includes the molecular composition of the cell and its structure, thus differentiating between cell types and their physiological states based on their full biochemical features^23^,^24^,^25^,^26^,^27^,^28^,^29^. Indeed, RS has been used recently as a novel technique to analyze precancerous tissues in the esophagus, colon, and cervix^30^,^31^,^32^,^33^, as well for the identification, classification, and diagnosis of many cancer types^34^,^35^,^36^,^37^,^38^,^39^,^40^.

In the present study, we demonstrate that RS can be used to identify and classify three B-leukemia cell types that closely mimic the different differentiation/maturation stages of B-ALL cells (i.e., RS4;11, REH, MN60 cells) *versus* their normal B-cell counterparts. We have also used RS to analyze the biochemical features of these B-leukemia cell lines after low-dose and noncytotoxic treatments with methotrexate (MTX) and 6-mercaptopurine (6MP), the two key drugs used in current B-ALL maintenance therapy^41^.

To identify specific RS peaks that correlate with the different stages of these B-leukemia cells, we defined the spectral changes after these B-ALL therapies in comparison with those seen under all-*trans*-retinoic acid (ATRA) treatment (used in the treatment of a different leukemia disease). Principal component analysis (PCA) was used to confirm the significance of these Raman spectral markers in the definition of the differentiation/maturation stages of B-ALL cells and in the detection of minimal residual disease. Finally, we applied the RS approach to discriminate between normal B-lymphocytes and B-lymphocyte-enriched fractions from patients with B-ALL.

## Results

### Identification and classification of acute lymphocytic leukemia cell

**Figure 1:**
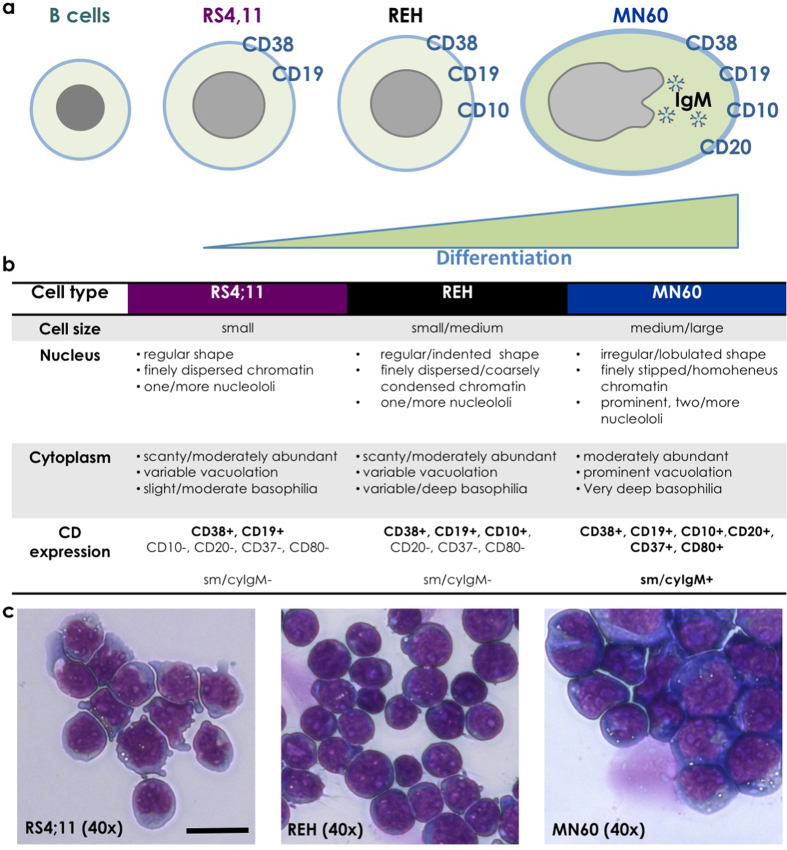
Differentiation scheme and morphology of the RS4;11, REH and MN60 B-leukemia cell lines. (**a**) Immunophenotypic profile with the coordinate and sequential well-characterized plasma-membrane antigen expression. (**b**) Morphologic features and immunophenotypic expression pattern. (**c**) Representative images of the cell morphology using May Grunwald-Giemsa histochemical staining for DNA and RNA molecules. Scale bar: 10 μm.

The first two cell models that we used were originally derived from patients with B-ALL: the RS4;11 and REH cell lines, which are both classified as the L2-blast (i.e., B-cell precursor leukemia) subtype^42^,^43^,^44^,^45^,^46^. The third cell model was the MN60 cell line, which represents a more differentiated B-ALL cell type, as it is classified as the L3-blast (i.e., B-cell leukemia) subtype^47^,^48^. To define these cell lines further, we initially carried out immunophenotypic and morphological characterization of the expression profiles of the sequential plasma-membrane antigens of these cell lines, as summarized in Fig. 1.

**Figure 2:**
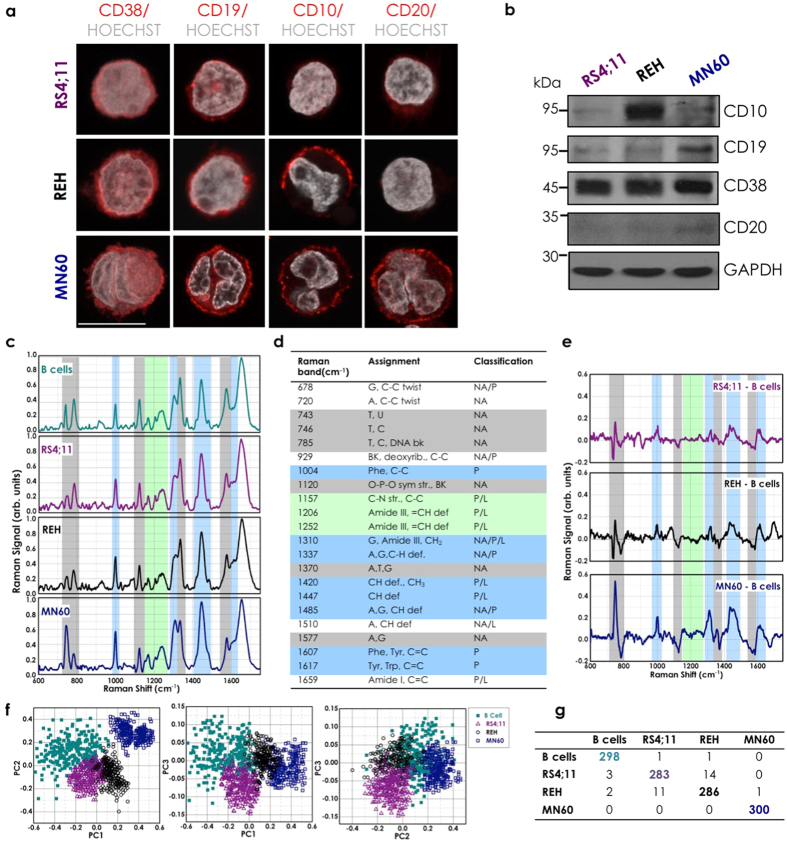
Identification and classification of acute lymphocytic leukemia cell. (**a**) Representative confocal microscopy images of RS4;11, REH and MN60 B-leukemia cells fixed and processed for immunofluorescence analysis with anti-CD38, anti-CD19, anti-CD10 and anti-CD20 monoclonal antibodies (red), to monitor their expression levels. Gray, Hoechst-33258 nucleic-acid staining. Scale bar: 10 μm. (**b**) Representative immunoblotting of RS4;11, REH and MN60 cells with antibodies against CD10, CD19, CD20 and CD38 (as indicated). Glyceraldehyde 3-phosphate dehydrogenase (GAPDH) is shown for the internal protein levels and molecular weight standards (kDa) are indicated on the left of each panel. The blots have been run under the same experimental conditions. (**c**) Mean Raman spectra of normal B-lymphocytes and the three analyzed B-leukemia cell lines (as indicated). Each spectrum is an average of 300 cells. (**d**) Raman spectral classification and assignment^22^,^23^. Abbreviations: def, deformation; str, stretching; bk, vibration of backbone; sym, symmetric; A, adenine; C, cytosine; G, guanine; T, thymidine; U, uracil (ring breathing modes of DNA/RNA bases); Phe, phenylalanine; Tyr, tyrosine; Trp, tryptophan; NA, nucleic acids, P, proteins; L, lipids. (**e**) Difference spectra obtained by subtracting the normal B cell mean Raman spectrum from the RS4;11, REH and MN60 B-leukemia cell mean Raman spectra (as indicated). (**f**) PCA scatter plots comparing control B-lymphocytes and B-leukemia cells. (**g**) Confusion matrix for the classification of the control B-lymphocytes and the B-leukemia cells.

Using the well-defined quadruple CD38/CD19/CD20/CD10 staining for leukemia cells^49^,^50^, we first examined these cells under confocal fluorescence microscopy and by Western blotting (Fig. 2a,b). CD38 and CD19 were expressed on the surface of the plasma membranes of all of these three cell lines, and their expression increased for the different differentiation stages from RS4;11 and REH cells to MN60 cells. CD20 was expressed only on the plasma membranes of the more differentiated MN60 cells^1^ (Fig. 1a,b). Finally, CD10 expression increased for the differentiation from RS4;11 to REH, and then decreased in MN60 cells, as already described for the L2-blast and L3-blast subtypes^1^. These immunophenotypic analyses validated the RS4;11 and REH cells as a model for the L2 subtype and the MN60 cells as a model for the L3 subtype of B-ALL cells.

To evaluate the RS signatures that might specifically identify differences in the biochemical compositions of these L2 and L3 B-leukemia cells *versus* their normal B-cell counterpart purified from human peripheral blood as described in the Methods and reported in Supplementary Fig. 1, we acquired Raman spectra from these RS4;11, REH and MN60 transformed B-leukemia cells and from B-lymphocytes (Fig. 2c)^22^. More than three hundred spectra were recorded and evaluated.

Specifically, all of the spectral signals were acquired taking into account four key experimental parameters. First, during acquisition, the laser was focused on the lymphocyte nucleus to limit the spectral variability from individual cells^51^ (see Supplementary Materials and Supplementary Fig. 2 on line). Second, for each cell type, the cells analyzed were derived from different cultures, to ensure that the observed differences were the result of biological variations rather than variations in instrument background or cell culture conditions^51^. Third, for each experimental session, more than 50 cells were analyzed, to take into account possible biochemical differences in the spectral data due to variations in cell-cycle progression^52^. Finally, the spectra that were obtained from cells with morphological alterations due to apoptotic status or that were in transition through the mitosis phase (visible and well defined under optical microscopy) were not included in the subsequent analyses.

The Raman spectrum of lymphocyte B cells (Fig. 2c) contained spectral features of DNA and RNA that arose from the individual bases (i.e., adenine, thymine, cytosine, guanine, uracil), as well as from the sugar-phosphate backbone of DNA (Fig. 2c, gray region; 700–800 cm^−1^, 1120 cm^−1^, 1370 cm^−1^, 1577 cm^−1^). The RS features of lipids arose from the stretching of the various CH_2_ and CH_3_ groups (Fig. 2c, green region; 1150–1250 cm^−1^). The RS features of proteins included contributions from the amide groups of the secondary protein structures (e.g., α-helices, β-sheets, random coils), aromatic amino acids (e.g., tryptophan, tyrosine, phenylalanine), and stretching or deformation of carbon atoms bonded with hydrogen, nitrogen, and other carbon atoms (Fig. 2c, cyan region; 1000 cm^−1^, 1300–1350 cm^−1^, 1400–1500 cm^−1^, 1600–1650 cm^−1^)^52^. The assignment of the Raman peaks is given in Fig. 2d^22^,^23^. To identify the most important spectral differences, the spectra of the B-leukemia RS4;11, REH, and MN60 cells were directly compared to those of the normal B-lymphocytes (Fig. 2c), and their spectral differences were calculated (Fig. 2e). This comparison revealed that these three transformed B-leukemia cell lines showed Raman spectra that were similar to the normal B-lymphocyte counterpart, although with significantly different mean intensities for some specific peaks. The spectra obtained showed negative differences around 785 cm^−1^, 1370 cm^−1^, and 1577 cm^−1^, which are due to the ring breathing modes in the DNA bases^22^,^23^,^40^ and near 1120 cm^−1^, which is due to the symmetric PO^2−^ stretching vibration of the DNA backbone^22^,^23^,^40^ (Fig. 2d,e). Additionally, the MN60 Raman spectra revealed higher intensity of the peak around 743 cm^−1^, which is generally assigned to RNA bases^22^,^23^,^36^ (Fig. 2d,e). Positive difference peaks were also seen at 1420–1485 cm^−1^ (CH and CH_2_ proteins/lipids), 1310 cm^−1^ (amide III band), 1337 cm^−1^ (CH proteins/nucleic acids), 1607–1617 (C=C vibrations), and 1650 cm^−1^ (amide I band)^22^,^23^,^36^,^40^,^53^,^54^ (Fig. 2d,e). It can also be noted that the magnitudes of these changes generally appeared more pronounced from the RS4;11 to the MN60 cells (Fig. 2e). Therefore, the intensity variations in these specific Raman peaks appear to correlate well with the differentiation/maturation stages of these B-leukemia cells.

### Principal component analysis for the Raman spectra

To define the efficiency and sensitivity of the proposed approach for single-cell cancer diagnosis and classification, we combined the RS analysis with a multivariate statistical method: PCA^54^. In the spectral range from 600–1800 cm^−1^, each Raman spectrum consists of 1200 variables at 1 cm^−1^ spacing; however, only a fraction of these contain useful information for this cell classification. PCA was performed on the spectral data to define the new dimensional space in which the major data variance can be detected and represented by a few relevant parameters, known as the principal components (PCs). These PCs form a model through which the RS signal of individual cells can be assigned a score. Therefore, PCA can provide a chart of the PCA scores of individually analyzed cells where similar Raman spectra cluster together^22^,^36^,^51^,^52^,^54^. Indeed, the combination of RS with several multivariate statistical methods has been successfully applied to discriminate between healthy and cancerous tissues, as well as between healthy and tumorigenic cells^22^,^36^,^38^,^51^,^52^.

Here, the first three PCs (PC1–3) accounted for close to 98% of the total variance, were used to generate scatter plots, where the clusters of points represented different cell groupings (Fig. 2f). The significance of these selected PCs was initially evaluated in an analysis of the loading values, where they showed the largest deviations from zero. This PCA analysis shows that when PC2 and PC3 were plotted against PC1, these three B-leukemia cell types and the normal B-lymphocyte counterpart formed four distinct and well-defined groups (Fig. 2f). The main result of this statistical analysis was then the creation of the confusion matrix that corresponds to the leave-one-out predictive classification for these normal and leukemia cells (Fig. 2g)^51^. Here, the diagonal values of this confusion matrix indicate that 1167 out of 1200 cases were classified correctly, which thus resulted in an efficiency of 97.3%.

### Analysis of acute lymphocytic leukemia regression through low-dose maintenance therapy

**Figure 3:**
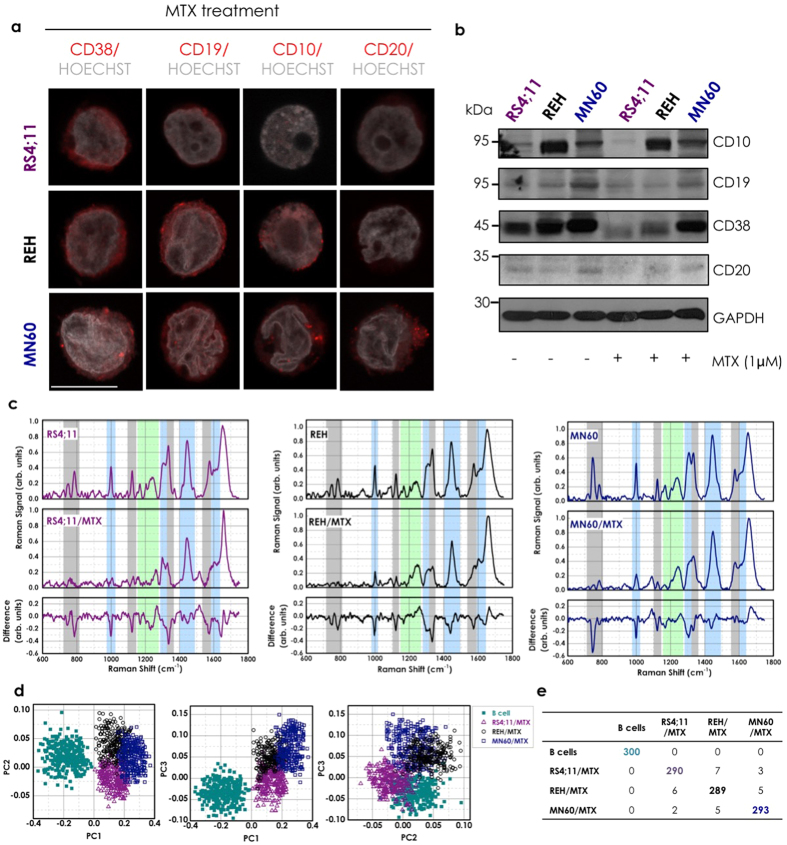
Acute lymphocytic leukemia regression through low-dose of MTX. (**a**) Representative confocal microscopy images of RS4;11, REH and MN60 B-leukemia cells treated with 1 μM MTX for 72 h to mimic the clinical B-ALL maintenance therapy. Cells were fixed and processed for immunofluorescence analysis with anti-CD38, anti-CD19, anti-CD10 and anti-CD20 monoclonal antibodies (red) to monitor their expression levels. Gray, Hoechst-33258 nucleic-acid staining. Scale bar: 10 μm. (**b**) Representative immunoblotting of RS4;11, REH and MN60 B-leukemia cells treated as in (**a)**, with antibodies against CD10, CD19, CD20 and CD38 (as indicated). Glyceraldehyde 3-phosphate dehydrogenase (GAPDH) is shown for the internal protein levels and molecular weight standards (kDa) are indicated on the left of each panel. The blots have been run under the same experimental conditions. (**c**) Raman spectra of three B-leukemia cell lines recorded without and with MTX treatment as in (**a)**. The difference spectra were obtained by subtracting the untreated from the treated spectra (bottom panels). (**d**) PCA scatter plots comparing the untreated and MTX-treated B-leukemia cells. (**e**) Confusion matrix for the classification of the untreated and MTX-treated B-leukemia cells.

**Figure 4:**
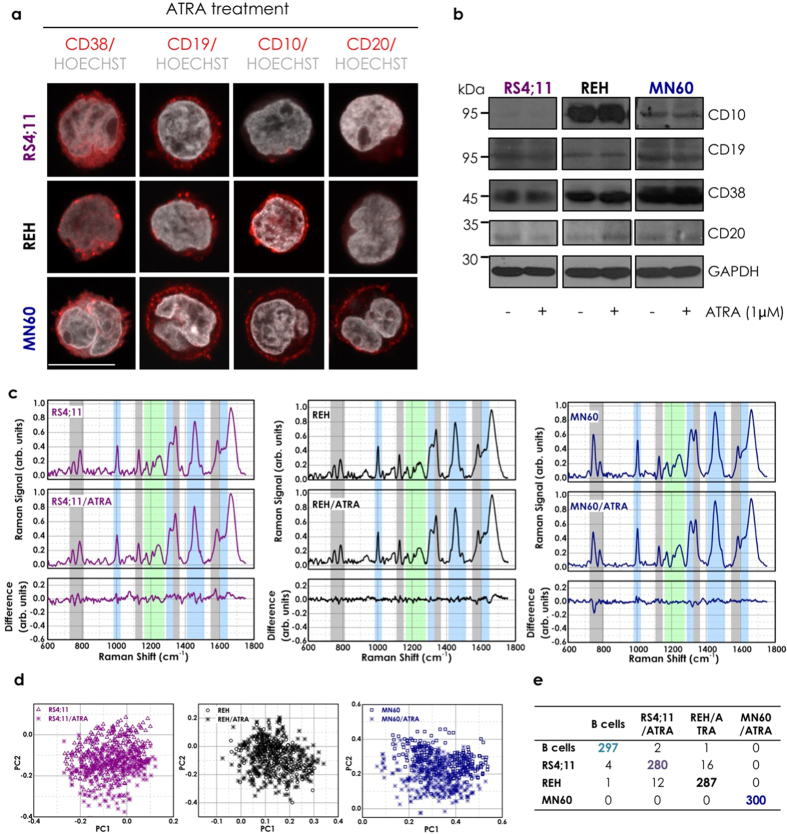
Effect of ATRA treatment on acute lymphocytic leukemia cells. (**a**) Representative confocal microscopy images of RS4;11, REH and MN60 B-leukemia cells treated with 1 μM ATRA for 72 h, and fixed and processed for immunofluorescence analysis with anti-CD38, anti-CD19, anti-CD10 and anti-CD20 monoclonal antibodies (red) to monitor their expression levels. Gray, Hoechst-33258 nucleic-acid staining. Scale bar: 10 μm. (**b**) Representative immunoblotting of RS4;11, REH and MN60 B-leukemia cells treated as in a, with antibodies against CD10, CD19, CD20 and CD38 (as indicated). Glyceraldehyde 3-phosphate dehydrogenase (GAPDH) is shown for the internal protein levels and molecular weight standards (kDa) are indicated on the left of each panel. The blots have been run under the same experimental conditions. (**c**) Raman spectra of three B-leukemia cell lines recorded without and with ATRA treatment as in. (**a**) The difference spectra were obtained by subtracting the untreated from the treated spectra (bottom panels). (**d**) PCA scatter plots comparing the untreated and ATRA-treated B-leukemia cells. (**e**) Confusion matrix for the classification of the untreated and ATRA-treated B-leukemia cells.

To provide a scenario as close as possible to the clinical setting, we analyzed these particular RS variations under ‘treatment’ with two clinically demonstrated beneficial drugs that are used in maintenance chemotherapy of patients with B-ALL: MTX and 6MP. Low doses of MTX and 6MP that provide patients with peak plasma concentrations range from 0.01 μM to 1 μM^41^ are used to induce specific B-ALL regression. MTX and 6MP are also specific treatments for patients with B-ALL, as they do not provide benefits for other leukemias, such as for regression of acute promyelocytic leukemia, where the maintenance therapy is primarily ATRA^41^. These three transformed B-leukemia cell lines were thus treated for 72 h with 1 μM MTX, 1 μM 6MP, or 1 μM ATRA (as the control)^41^. The cells were then analyzed by immunofluorescence microscopy and Western blotting to determine whether the B-ALL regression obtained in patients under similar conditions is associated with decreased expression of particular B-leukemia antigens. This MTX treatment significantly reduced the plasma membrane staining of CD38 and CD19 in all of these B-leukemia cell lines, along with that of CD10 in the REH and MN60 cells, and of CD20 in the MN60 cells (Fig. 3a versus non-treated control cells shown in Fig. 2a). These immunofluorescence data were supported and confirmed by Western blotting (Fig. 3b). Moreover, reduced intensity of nucleic-acid fluorescence staining was also observed in all three of these MTX-treated B-leukemia cell lines (Fig. 3a versus non-treated control cells shown in Fig. 2a). Similar data for both antigen expression and nucleic acids (stained with Hoechst dye) were obtained with 6MP treatment (data not shown), while no effects were detected with ATRA treatment (Fig. 4a,b). Flow Cytometry analysis confirmed confocal microscopy study regarding the down-regulation of CD19, CD10 and CD38 differentiation antigens upon induction with MTX, while no effects were detected with ATRA treatment, as shown in Supplementary Fig. 3. These data indicate that MTX treatment specifically reverted the B-cell differentiation process for these L2 (RS4;11 and REH cells) and L3 (MN60 cells) subtypes of B-leukemia cells (Fig. 3a,b), while as expected, the ATRA acute promyelocytic leukemia treatment had no effect (Fig. 4a,b). Of note, under these conditions, no drug-mediated apoptotic effects were detected, as indicated by the nucleolus and chromatin staining with the Hoechst 33258 dye (Figs 3a and 4a)^55^.

Figure 3c shows the comparison of the mean Raman spectra of these B-leukemia RS4;11, REH, and MN60 cells before and after the MTX treatment, and the difference spectra obtained by subtraction of the MTX-treated spectra from the untreated cell spectra. The MTX-treated cells showed lower Raman intensities of the peaks related to nucleic acids, as can be seen at 785 cm^−1^, 1120 cm^−1^, 1370 cm^−1^, and 1577 cm^−1^. These reductions in the Raman bands suggest that the levels of nucleic acids are lower after the chemotherapy in these B-leukemia cells. Interestingly, the Raman spectra of the 6MP-treated B-leukemia cells showed similarly lower Raman intensities of the peaks related to nucleic acids as seen for the MTX-treated cells (Supplementary Fig. 4). As well as these peaks, the MTX-treated and 6MP-treated cells showed lower peak heights in the bands at 1004 cm^−1^ (CC ring breathing mode of phenylalanine) and 1447 cm^−1^ (CH_2_ deformation mode of proteins). The intensity of 1447 cm^−1^ Raman band is insensitive to the protein structure and depends only on the number of proteins, which indicated that these MTX and 6MP treatments induced specific reductions in the cell protein content^56^. Conversely, the ATRA-treated cells did not show reduced expression of CD10, CD19, CD20, and CD38 at the plasma membrane and nucleic-acid fluorescence staining intensity (Fig. 4a,b), and in turn, did not show significant modifications in the Raman spectral region related to protein-DNA ratio (around 1447 and 785 cm^−1^; Fig. 4c). In general, the difference spectra between the ATRA-treated and nontreated B-leukemia cells show only slight spectral variations.

Finally here, we used PCA to evaluate the discrimination between the drug-treated *versus* the normal B cells according to the RS. Figure 3d shows the PCA scatter plots of the scores of PC1-3 of 300 of each of the control B cells and MTX-treated B-leukemia RS4;11, REH and MN60 cells. The control B cells can be easily distinguished from the MTX-treated B-leukemia cells by this PCA, as shown by the distinct group of the B-lymphocytes. The data reported in the confusion matrix (Fig. 3e) indicate that 1186 out of 1200 of these cells were correctly classified, which gives an efficiency of 98.8%. Similar data were obtained with the 6MP-treated leukemia cells. On the other hand, by comparing the Raman spectra of the B-leukemia cells to those of the ATRA-treated B-leukemia cells using PCA, there was large overlap according to the PC1 *versus* PC2 plots, and thus little, if any, effects of ATRA on these PCs (Fig. 4d).

### Proof of principle with clinical samples

We then extended the present study to clinical samples, with the analysis of the Raman spectra of B-cell–enriched lymphocyte fractions purified from peripheral blood of three patients with B-ALL.

**Figure 5:**
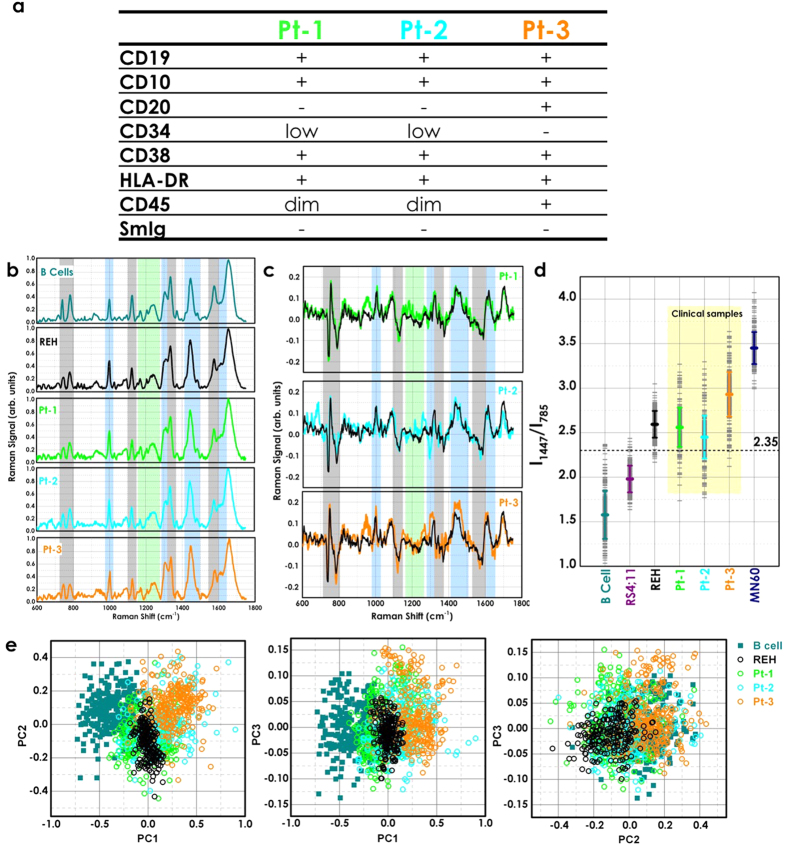
Proof of principle with clinical samples. (**a**) Immunophenotypes of the B-ALL cells from patients. The Pt-1 and Pt-2 B-ALL cells show a ‘common’ B-ALL immunoprofile due to the surface expression of CD19, CD10, CD38, CD45 (intermediate expression level, dim) and HLA-DR, with low surface expression of CD34 (low) and absence of CD20, and surface Immunoglobulins (SmIg). The Pt-3 B-ALL cells were more differentiated than those from Pt-1 and Pt-2, due to the surface membrane presence of CD20 with CD19, CD10, CD38 and HLA-DR, as well as bright intensity of expression for CD45. (**b**) Mean Raman spectra from 300 acquisitions of normal B-lymphocytes, REH B-leukemia cells, and the three clinical B-ALL cell samples. (**c**) Difference spectra obtained by subtracting the normal B-lymphocytes Raman spectrum from the clinical B-ALL samples and the REH B-leukemia cell spectra (black line). (**d**) Plot of the intensity ratios of the Raman signals at 1447 cm^−1^ and 785 cm^−1^ (I_1447/I_785), as indicated. (**e**) PCA scatter plots comparing normal B B-lymphocytes, REH B-leukemia cells, and the three clinical B-ALL cell samples.

First, we analyzed these leukemic cells using standard multi-parametric flow cytometry to characterize them based on the immunophenotypic classification (Fig. 5a). These leukemic cells from patients 1 (Pt-1) and 2 (Pt-2) showed very similar immunological features, including: plasma-membrane expression of CD19, CD10, CD38, and CD45 (intermediate expression level), and of HLA-DR antigens, with low surface expression of CD34 and no surface expression of CD20, and of immunoglobulins (SmIg) (Fig. 5a). According to this antigenic profile, these leukemia cells of Pt-1 and Pt-2 can both be classified as ‘common B-ALL’ and thus they should have similar Raman spectra to the REH B-leukemia transformed cell line. Conversely, for the Pt-3–derived cells, in addition to positivity for CD19, CD10, CD38, CD45, and HLA-DR, there was also plasma-membrane expression of CD20, which indicated that their B-ALL was of a more differentiated type than for the Pt-1 and Pt-2 cells (Fig. 5a). However, these Pt-3–derived cells did not express surface or cytoplasm immunoglobulins (Fig. 5a), which indicated that they might have originated from B-cell differentiation/maturation blocked at an intermediate process between the pro-B and the pre-B maturation stages. Thus, according to this immunological feature, the leukemic cells from Pt-3 should have a Raman spectrum with a profile in-between that of the B-leukemia REH and MN60 transformed cell lines.

Figure 5b shows the Raman spectra of normal B cells, the B-leukemia REH cells, and those acquired from the three cell samples from the patients with B-ALL. The mean Raman spectra of Pt-1 and Pt-2 cells appear to be similar to each other and similar to the Raman spectrum of the REH cells. Conversely, as illustrated in Fig. 5c, there were distinct differences between the Raman spectrum of normal B cells and those of the three patient B-ALL cell samples. The peaks most exclusively related to the ring breathing modes in DNA bases (i.e., 785, 1120, 1370, 1577 cm^−1^) were significantly reduced in intensity in the Raman spectra of Pt-1 and Pt-2, while the CH_2_ deformation mode at 1447 cm^−1^ was significantly stronger (Fig. 5c). These differences were more pronounced in the Pt-3 Raman spectrum, as this B-ALL cell sample was more differentiated from the previous two.

To clarify the biochemical classification and spectral variations for the B-leukemia cells, we used an empirical analysis based on the intensity ratio of two prominent Raman bands, at 1447 cm^−1 ^and 785 cm^−1^ (I_1447_/I_785_). Figure 5d shows the plot of the I_1447_/I_785_ ratios according to the cell types. The mean (±s.d.) of the ratio for the normal B cells (R_Bcell_ = 1.6 ± 0.3) was significantly different from the means of the B-leukemia cells (R_RS4;11_ = 1.98 ± 0.17; R_REH_ = 2.59 ± 0.18; R_MN60_ = 3.45 ± 0.20) and the patient-derived B-ALL cells (R_Pt−1_ = 2.56 ± 0.27; R_Pt−2_ = 2.45 ± 0.25; R_Pt−3_ = 2.93 ± 0.27). These differences reflected the relative changes in the potential biological markers from cell surface antigens, cytoplasmic proteins, and DNA content^56^. Additionally, the spectral variability measured in the clinical samples was much higher compared to the B-leukemia cell lines. The overall spectral intensities varied by 20% about the mean for normal B cells, by 7–8% for the three B-leukemia cell lines, and by 10–12% for the clinical samples. The nonparametric intensity ratio provided a diagnostic sensitivity of 98.7% and specificity of 95.7% for separating the normal B cells and B-leukemia REH cells (P < 0.00001). With the clinical samples, the measured diagnostic sensitivity and specificity for the intensity ratios and relative to the normal B cells were 79% and 81% for patient 1, 80 and 83% for patient 2, and 87 and 85% for patient 3, respectively.

We also used PCA for classification of these clinical samples. These data for the normal B cells, the REH B-leukemia cells, and the three patient samples are shown in the scatter plots of Fig. 5e. The PCA algorithm achieved identification of each pathological clinical sample with sensitivities of 88, 89 and 94% for Pt-1, Pt-2 and Pt-3, respectively, and specificities of 85, 88 and 90%, again for Pt-1, Pt-2 and Pt-3, respectively, which provides 5–10% improvements compared with the empirical methods using the intensity ratios. Therefore, although the spectral variability in the clinical samples is more pronounced compared to the B-leukemia cell lines, analysis of the Raman spectra allows the discrimination of the B-ALL clinical cell samples of these patients from the normal B cells with high sensitivity and specificity.

## Discussion

This study demonstrates the potential application of RS to patients with B-ALL for cell identification and classification based on the B-cell differentiation/maturation stage. We used three transformed B-leukemia cell lines, RS4;11, REH and MN60 cells, that are considered to be useful model systems to study the pathophysiology of hematopoietic tumors^1^,^46^.

As these three cell lines belong to the human B-lymphoid lineage, their Raman spectra appeared very similar. However, these RS4;11 and REH B-leukemia cells are representative of the L2 B-ALL subtype, and the MN60 B-leukemia cells are representative of the L3 B-ALL subtype, and their spectra show subtle differences in intensities of specific defined Raman peaks. For instance, the relative peak intensities at 785 cm^−1^, 1120 cm^−1^, 1370 cm^−1^, and 1577 cm^−1^ (related to DNA bases) were lower in these B-leukemia cells compared to normal B-lymphocytes, which indicates a reduction in the nucleic-acid content of the cells. This effect might be due to breaks in and translocations of several chromosomes (e.g., in REH cells, the X-chromosome is completely missing^57^) or to chromatin decondensation^58^. Of note, the Raman spectrum of the MN60 B-leukemia cells was slightly different from those of both the RS4;11 and REH B-leukemia cells, which also confirmed that these MN60 B-leukemia cells can be classified as a distinct B-leukemia cell subtype, as indeed is the case based on its more differentiated maturation stage. The Raman spectrum for the MN60 B-leukemia cells showed greater intensity of the peak around 743 cm^−1^, which would appear to be due to an increase in ribosomal RNA content, which in turn correlates with higher synthesis and content of proteins. Indeed, the morphological assessment of these MN60 B-leukemia cells showed intense cytoplasmic staining that is indicative of the presence of large amounts of ribosomal RNA in the rough endoplasmic reticulum where the biosynthesis of immunoglobulins and other proteins takes place^45^,^59^. In addition, there were relative increases in the Raman spectra intensities for the bands related to amide III (1310 cm^−1^), amide I (1650 cm^−1^), and CH/CC (1337, 1420–1485, 1607–1617 cm^−1^) proteins, which correlated with the protein structure in these three B-leukemia cell lines. The magnitudes of these peaks increased from the RS4;11 cells to the MN60 cells, in agreement with the extent of expression of plasma-membrane antigens, nucleus/cytoplasm ratio and cytoplasmic immunoglobulins during the B-cell differentiation/maturation process. Therefore, the distinctive differences in the Raman spectra between the normal B-lymphocytes and the B-leukemia cells suggests that RS can be used to reveal molecular changes associated with these pathological transformations.

We also explored PCA here, together with a leave-one-out cross-validation approach, for B-leukemia cell identification and classification^50^, which provided a diagnostic efficiency of 97.3% for separating B-leukemia *versus* normal B-lymphocytes. The PCA also indicated larger spread of the normal B cell cluster compared to the populations of B-leukemia cells, which was probably due to greater cell-to-cell variation for the different donors of the normal B-lymphocytes. Additionally, the MN60 cluster was completely separated from the others, which again confirms that this model system is more differentiated. Notably, only two of 300 control cells were predicted incorrectly, and five of the B-leukemia cells were incorrectly assigned to the normal B-lymphocyte group, and therefore the risk of false positives with this model appears to be minimal.

Detection and classification of leukemia cells is only the first step in the clinical management of these patients. Constant cell monitoring of the chemotherapy effect holds great potential for improving treatment strategies and might be crucial for the detection of minimal residual disease. For this purpose, we focused the present study on the maintenance therapies for patients with B-ALL, with the aim to suppress the drug-resistant population of B-leukemia cells^1^,^41^. Using independent and complementary approaches, as immunofluorescence, Western blotting and RS, we identified some biological features that were modified under these B-ALL specific maintenance therapies. Of note, low-dose treatments were used here to better analyze the accuracy of our approaches for the detection of these specific biochemical changes. In more detail, under both MTX and 6MP treatments, all three of these B-leukemia cell lines showed B-leukemia regression through the reversing of the B-cell differentiation/maturation process, which was identified and classified according to the specific antigen expression profiles, and promoted changes in the nucleic acid contents without any apoptotic effects. These specific modifications should be explained by the mechanisms of action of the chemotherapeutic drugs used in this study. The reduced nucleic acid content can be explained by the inhibition of the enzyme dihydrofolate reductase through MTX-derived metabolites^60^. The dihydrofolate reductase inhibition arrests the synthesis of both purines and thymidine, which alters DNA replication and RNA synthesis, and thus, in turn, the nucleic-acid cell content. Similarly, the mechanism of action of 6MP consists of inhibition of the *de-novo* pathway for purine ribonucleotide synthesis^61^ that is required for DNA repair, methylation, and mitotic duplication. On the other hand, the reduced protein content can be explained by the decreased expression of plasma membrane antigens (e.g., CD10, CD19, CD20, CD38) and cytoplasmic immunoglobulins. Interestingly, both MTX and 6MP treatments similarly affected the Raman spectra of these three B-leukemia cells, with reduced intensities of peaks related to DNA, RNA and protein content. To demonstrate the specific ability of this RS approach for the detection of the anti-leukemic effects of MTX and 6MP treatments on these B-leukemia cells we used ATRA treatment as a control. The Raman spectra of the ATRA-treated and nontreated B-leukemia cells did not show any significant differences. Indeed, the relative variations in the Raman band intensities were much smaller than those observed between normal B-lymphocytes and the B-leukemia cells, or between MTX-treated or 6MP-treated and nontreated B-leukemia cells. Additionally, the PCA scatter plots and the cross-validation data demonstrate that although the MTX-treated (or 6MP-treated) B-leukemia cells showed reduced expression of plasma membrane antigens and DNA, their spectra can be separated from the normal B-lymphocytes with an efficiency of about 99%. These findings confirm that very specific Raman markers can be used to discriminate normal from both leukemia cells and leukemia cells under maintenance treatment, which suggests that RS can be used in the detection of minimal residual disease.

Finally, we extended the Raman approach to three clinical patient samples. Pt-1 and Pt-2 had the preliminary classification of ‘common B-ALL’, and Pt-3 had B-ALL derived from malignant transformation of a B-cell progenitor that was intermediate between the pro-B and pre-B maturation stages. The distinctive differences in the Raman spectra between the normal and the clinical samples confirmed and further reinforced these observations.

To develop simple but effective algorithms for differentiating B-leukemia cells from normal B-lymphocytes, the nonparametric empirical approach that uses peak intensity ratio measurements of specific Raman bands has been widely applied to evaluate malignant changes in several studies^62^,^63^. Here, we show that the nonparametric intensity ratios of the two prominent Raman bands at 1447 cm^−1^ (CH_2_ mode proteins) and 785 cm^−1^ (ring breathing modes in DNA bases), I_1447_/I_785_, provided good diagnostic sensitivity (79–87%) and specificity (81–85%). The significant differences in the intensity ratios between the normal B-lymphocytes and the clinical patient B-ALL samples might reflect the relative changes in the concentrations of potential biological markers, as cell surface antigens, and cytoplasmic protein and DNA contents. Additionally, despite the higher variability in the clinical patient B-ALL samples compared to the three B-leukemia cell lines, this Raman approach allowed significant discrimination between normal B-lymphocytes *versus* these B-ALL cells.

This relatively simplistic empirical analysis only used a limited number of Raman peaks, and most of the information contained in the Raman spectra is not included. Therefore, we also performed PCA with these clinical samples. From this PCA analysis, several considerations arose: (i) the spectral variability in these clinical samples was more pronounced compared to the B-leukemia cell lines; (ii) the REH B-leukemia cell data fell within a smaller region defined by the B-ALL cells from Pt-1 and Pt-2; (iii) the data for the B-ALL cells from Pt-3 showed better demarcation from normal B-lymphocytes, and thus these B-ALL cells from Pt-3 were more differentiated than those from Pt-1 and Pt-2, and showed an intermediate behavior to that of the REH and MN60 B-leukemia cell lines; and (iv) RS can be used to differentiate between normal B-lymphocytes and clinical samples from patients with B-ALL with high sensitivity and specificity. In more detail, the PCA algorithm achieved identification of each pathological clinical sample with a sensitivity from 88–94% and a specificity from 85–90%. These sensitivity and specificity data in this diagnosis provided by RS can now set the stage for more specific clinical studies.

In conclusion, this study shows that RS and PCA allow differentiation between normal B-lymphocytes and B-ALL cells at different maturation stages, which has important implications for clinical practice. The feature-rich and specific Raman spectra offers the possibility of highly multiparameter measurements that could represent a major step forward towards the realization of a non-destructive, label-free Raman-based flow cytometer for blood cell identification. Further studies with both clinical samples before and after chemotherapy treatment will still be needed to understand the potential limitations of this RS approach.

## Methods

### Ethics statement

All studies on the healthy volunteers and the patients were performed in accordance with the approved guideline of the IRCCS SDN. Informed consent for the use of the biological samples for research purposes was obtained from both the healthy volunteers and the patients enrolled in this study. The experimental protocol was approved by the Ethics Committee of the IRCCS SDN (Comitato Etico Fondazione SDN approved on 21.09.2012).

### Cell culture and drug treatments

The RS4;11, REH and MN60 B-leukemia cell lines were obtained from Leibniz Institute DSMZ-German Collection of Microorganisms and Cell Cultures (Germany). The RS4;11 cell line (an L2 subtype) was originally derived from a 32-year-old female patient with B-ALL at relapse^42^,^45^ (see also Fig. 1). The REH B-leukemia cell line was originally derived from a 15-year-old male patient with common B-ALL at relapse, and these are morphologically similar to the RS4;11 B-leukemia cells^44^. The MN60 B-leukemia cell line (an L3 subtype) was originally derived from a 20-year-old male patient with B-ALL during remission phase^45^. The RS4;11 and MN60 B-leukemia cell lines^42^ were maintained in α-MEM supplemented with 10% (v/v) fetal bovine serum, 2 mM L-glutamine, 50 U/mL penicillin and 50 μg/mL streptomycin. The REH B-leukemia cell line^62^ was maintained in RPMI supplemented with 20% (v/v) fetal bovine serum, 2 mM L-glutamine, 50 U/mL penicillin and 50 μg/mL streptomycin. For the drug treatments, a 10 mM stock solution of 6MP in 1 M NaOH and 10 mM stock solutions of MTX and ATRA in dimethylsulfoxide were diluted in cell cultured medium, and the cells were treated for 72 h before processing for immunofluorescence or Western blotting. Note that the final concentration of dimethylsulfoxide in these treatments was at least 100-fold below the described differentiating concentration for dimethylsulfoxide^64^.

### Clinical cell samples

Normal B-cell-enriched fractions of lymphocytes were obtained using EasySep Negative selection kits (Stemcell Technologies Inc), for heparinized venous blood obtained from healthy volunteers, as previously described^65^, and according to the manufacturer instructions (see Supplementary Materials).

B-leukemia cells were collected from the peripheral blood of three male patients with B-ALL (Age 22-, 5 and 23-years old for Pt-1, Pt2 and Pt3, respectively). Blood samples for all patients were withdrawn at diagnosis, prior to therapeutic regimen start.

### Immunofluorescence microscopy

Confocal images were acquired and analyzed using an inverted confocal microscope system (Zeiss LSM700; Carl Zeiss) with a 63× oil-immersion objective with a resolution of 1024 × 1024 pixels, with the files exported as Tiff files. The images were cropped and optimized for brightness and contrast with Photoshop, and composed using Illustrator (Adobe Systems).

### Western blotting

The RS4;11 (6 × 10^4^), REH (1 × 10^5^), and MN60 (3 × 10^4^) cells were suspended in 2 mL cultured medium in 6-well plates and left for 72 h. These were then lysed with 100 μL Laemmli buffer. The total protein was separated on 10% SDS-PAGE, transferred onto nitrocellulose, and subjected to Western blotting (see Supplementary Materials).

### Raman spectroscopic measurements and spectral processing

Data were acquired using a RS system as previously described^66^,^67^. Using a water-immersion objective lens (UPLSAPO 60XW, 1.2 NA; Olympus) and setting the entrance slit of the monochromator at an aperture of 250 μm, the spatial resolution of the Raman microscope was excitation spot was about 0.9 μm in diameter, and light diffusion in the cell resulted in a sampled volume of about 2.5 μm^3^. During data acquisition, the laser beam was focused on the cell nucleus. However, the scattered light contained average information from different cell sub-compartments (i.e., nucleus, cytosol, membrane). The average laser excitation power on the sample was 2 mW, to avoid cellular photodamage. Raman spectra were acquired with a 20-s integration time and a spectral resolution of 1 cm^−1^. To be suitable for Raman analysis, the cells were transferred to a quartz slide and the spectra measured within 1 h of the removal of the culture medium. A Matlab script was used to align the spectra to compensate for any small drift in the laser wavelength over the experimental period. The Raman spectra were background corrected by subtracting a third-order polynomial fit. The data were finally normalized with respect to the highest peak (Raman band at 1659 cm^−1^). For data analysis, a program developed in the Matlab platform that was based on PCA was used (see Supplementary Materials). A non-parametric diagnostic algorithm based on peak intensities was used for cell classification^62^,^63^,^67^.

### Statistical analysis

For data analysis, a program developed in the Matlab platform based on PCA was used. PCA is a multivariate technique that operates in an unsupervised manner and is used to analyze the inherent structure of the data^54^. The PCs correspond to linear combinations of the original variables, which are orthogonal to each other and designed in such a way that each one successively accounts for the maximum variability of the dataset. When the PC scores are plotted they can reveal relationships between samples (groupings). The leave-one-out method was used for cross validation of the model. In this method, we performed PCA of the whole dataset without one spectrum. The resultant model was used to predict the classification of the left-out sample spectrum. This cross-validation approach was used for each spectrum in the set, and we constructed a confusion matrix that summarizes the correct and incorrect spectra classifications^36^. Each row of the confusion matrix gives the predicted classification for a specific cell type. The diagonal terms of the confusion matrix give the number of correct predictive classifications, and by taking into account the mean of these values, it is possible to obtain information on the efficiency of the method.

## Additional Information

**How to cite this article**: Managò, S. *et al*. A reliable Raman-spectroscopy-based approach for diagnosis, classification and follow-up of B-cell acute lymphoblastic leukemia. *Sci. Rep*. **6**, 24821; doi: 10.1038/srep24821 (2016).

## Supplementary Material

Supplementary Information
