# Supplementary material for: A reliable Raman-spectroscopy-based approach for diagnosis, classification and follow-up of B-cell acute lymphoblastic leukemia
Source: Sci Rep. 2016 Apr 19;6:24821. doi: 10.1038/srep24821 (PMC4835730; doi:10.1038/srep24821)
Supplement: Supplementary Information [file srep24821-s1.pdf]

Supplementary Information

A reliable Raman-spectroscopy-based approach for diagnosis, classification and follow-up of B-cell acute lymphoblastic leukemia

Stefano Managò[1], Carmen Valente[1,*], Peppino Mirabelli[2], Diego Circolo[1], Filomena Basile[3], Daniela Corda[1] & Anna Chiara De Luca[1,*]

Results and discussion

B-ALL morphological characterization. Thin-layer of the RS4;11, REH and MN60 B-leukemia cells for morphological analysis were prepared using a cytocentrifuge (Thermo Scientific Shandon Cytospin4; Thermo Fisher Scientific Inc, USA) according to manufacturer instructions, and then stained using the May-Grunwald-Giemsa staining protocol (SIGMA-Aldrich, St. Louis, MO, USA). The cells were then examined under light microscopy at 40× magnification for the morphological analysis. The RS4;11 and REH B-leukemia cells showed small to medium nuclear size, with a regular shape, finely dispersed chromatin, and one or more nucleoli. The cytoplasm was scanty, with slight or moderate staining and variable vacuolation. Instead, the MN60 B-leukemia cells showed medium to large nuclear size, with an irregular and lobulated shape, finely stippled chromatin, and two or more prominent nucleoli. There was moderately abundant cytoplasm, with prominent vacuolation and very deep staining, typical of the L3 subtype B-ALL cells (see Fig. 1).

B-cell assessment of purity. The purity of the B-cells was measured using flow cytometry after the cells had been stained with a fluorochrome-conjugated antibodies mix composed of: anti-CD45 (pan-leucocyte marker) conjugated with fluorescein isothiocyanate; anti-CD56 (natural killer cell marker) conjugated with Rodamine-1; anti-CD19 (B-cell marker) conjugated with phycoerythrin-Texas Red conjugate; and anti-CD3 (T-cell marker) conjugated with phycoerythrin-cyanine5 tandem. These antibodies were from Beckman-Coulter. The fluorescence intensities were measured with a flow cytometer (FC-500; Beckman-Coulter, Milan, Italy), according to the manufacturer instructions. Forward scatter and side scatter gates were established to exclude dead cells and cell debris. The percentage of B-cells (CD19+/CD45+), T-cells (CD3+/CD45+) and NK-cells (CD56+/CD45+) were assessed before the immunomagnetic procedure, as shown in Supplementary Fig. 1 a, c and e, respectively. The percentage of B-cell enrichment was verified after the immunomagnetic separation procedure, according to Supplementary Fig. 1b. Contamination by T-cells and NK-cells was also determined (Supplementary Fig. 1d and f, and it was always negligible (<1.0%).

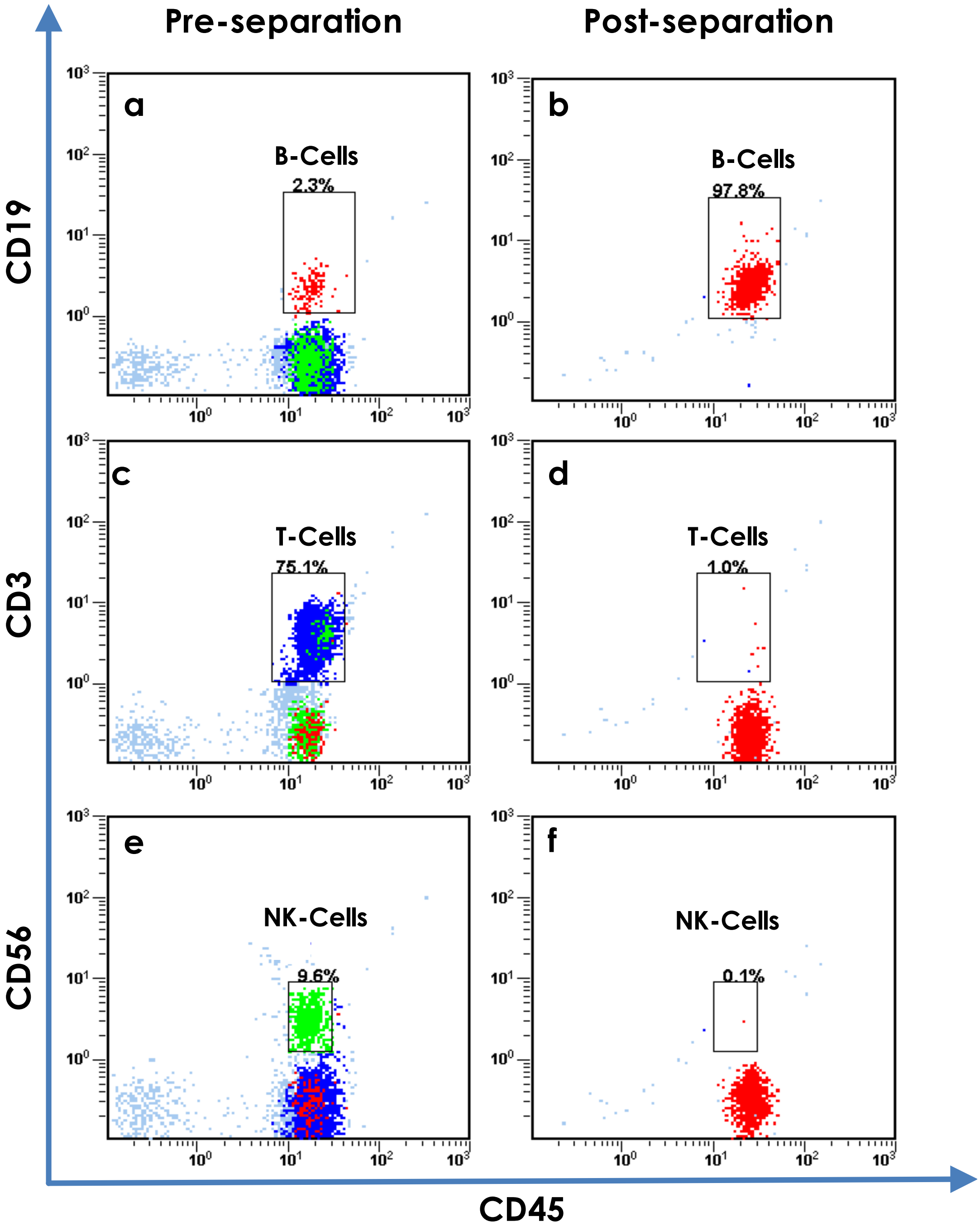

Supplementary Figure 1| Dot plots for the determination of human B-lymphocyte purification by flow cytometry. The percentage of B-cells (**a**, **b**, CD19+/CD45+, red events), T cells (**c**, **d**, CD3+/CD45+, blue events), and natural killer (NK) cells (**e**, **f**, CD56+/CD45+, green events) determined by flow cytometry, as pre-separation and post-separation through negative immunomagnetic selection. The content of the B-lymphocytes in the purified enriched fraction was routinely about 98% (as indicated) in all of the samples analyzed.

Reproducibility of Raman spectra. Spectra obtained from nucleus, cytoplasm and membrane of the studied cells (B-cells and RS4;11, REH and MN60 B-leukemia cells) were preliminary analyzed. Supplementary Figure 2 shows MN60 nucleus, cytoplasm and membrane spectra. The spectra have been calculated by acquiring three different spectra from the nucleus, cytoplasm and membrane within a cell and repeating the measurements on 20 different cells. The three spectra contain specific features for those regions of the cells, providing an intrinsic check for the region inside a cell from where the spectrum was obtained. However, no qualitative differences were observed between spectra obtained from different cells or different nucleus, cytoplasm and membrane regions within a cell. The position of the laser, during acquisition, was focused on the cell nucleus as this is where the most pertinent information is likely to originate, although with large volume, the system should also partially examine the membrane and cytoplasm above and below the nucleus.
In the nuclear region slightly differences (about 5%) in the intensity ratio I_{1447}/I_{785} (representative of protein-DNA ratio) were found (see Supplementary Figure 2).

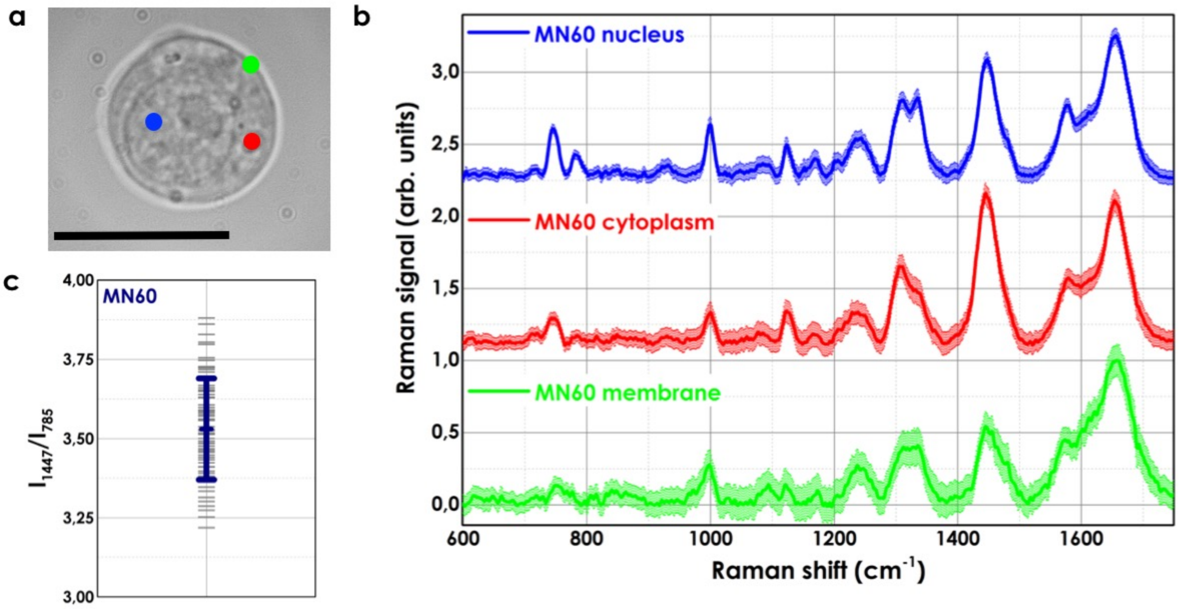

Supplementary Figure 2| Raman spectra reproducibility. (**a**) Representative optical image of MN60 B-leukemia cell. The Raman probe location during the spectra acquisition is also shown. Scale bar:10 µm. (**b**) Mean Raman spectra of nucleus, cytoplasm and membrane of MN60 B-leukemia cells. Each spectrum is an average of 60 acquisitions. (**c**) Intensity ratio of the Raman signals at 1447 cm^{-1} and 785 cm^{-1}.

Proportions of RS4;11, REH, and MN60 cells before and after treatment by flow cytometry (FCM) examination. FCM analysis confirmed confocal microscopy study regarding the down-regulation of CD19, CD10, CD38 and CD20 differentiation antigens upon induction with MTX, as shown in Supplementary Figure 3.

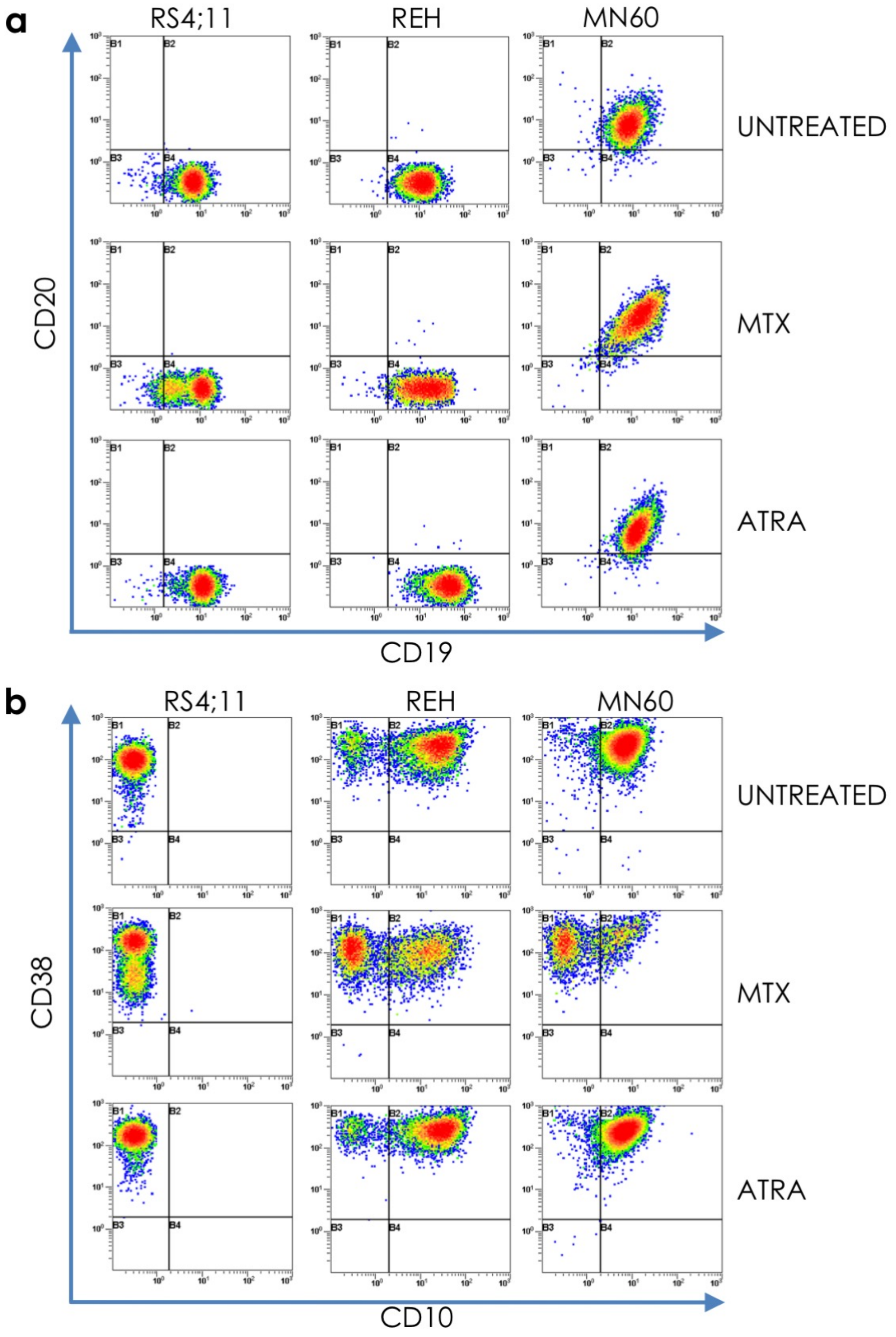

Supplementary Figure 3| Flow cytometry analysis of RS4;11, REH and MN60 cell lines. Panel A shows dot-plots analysis for the CD20 and CD19 surface expression on RS4;11, REH and MN60 cell lines in untreated conditions (Panel A, Upper Line), or treated for 72h with 1 µM of MTX (Panel A, middle line), or treated for 72h with 1 µM of ATRA (Panel A, bottom line). Panel B displays dot-plots analysis, for the CD38 and CD10 surface expression on RS4;11, REH and MN60 cell lines treated as in panel A.

Acute lymphocytic leukemia regression under 6MP-mediated treatment. The three different pathological cell lines were incubated with 1 µM 6MP for 72 h. The 6MP-mediated effects were studied using three different and parallel approaches: immunofluorescence microscopy, Western blotting, and RS. Supplementary Figure 4 shows the mean Raman spectra of the three B-leukemia cell lines recorded without and with 6MP treatments. The difference spectra obtained by subtracting the B-leukemia cell spectra from the 6MP-treated B-leukemia cell spectra are also shown. In all of the three B-leukemia cell lines, the spectra revealed reproducible differences in the spectral profiles that were characterized by: (i) reduction of Raman peaks at 780 cm^{-1}, 1089 cm^{-1}, 1370 cm^{-1}, and 1577 cm^{-1}, which suggested a reduced amount of nucleic acids under 6MP treatment; (ii) reduction of Raman peaks at 1000 cm^{-1} (C-C ring breathing of phenylalanine) and 1446 cm^{-1} (CH2 bending mode of proteins), related to protein structure, suggesting a reduced protein content in these 6MP-treated B-leukemia cells.

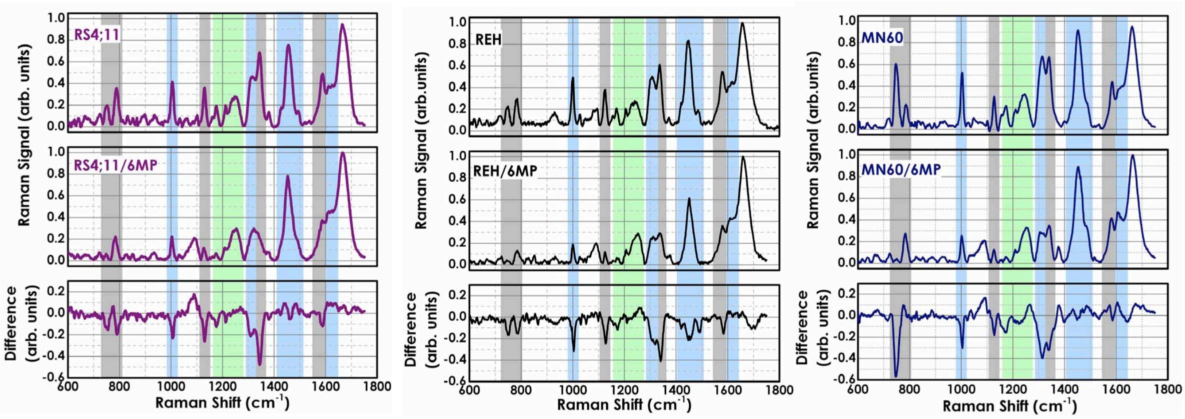

Supplementary Figure 4| Acute lymphocytic leukemia regression through low-dose of 6MP. Raman spectra of the three B-leukemia cell lines recorded without and with 1 µM 6MP for 72 h. The difference spectra obtained by subtracting the B-leukemia cell spectra from the 6MP-treated B-leukemia cell spectra are also shown (bottom panels).

Methods

Reagents. L-glutamine, penicillin, streptomycin, BSA, saponin, MTX, 6MP and ATRA were from Sigma Aldrich. Fetal bovine serum was from Gibco. Paraformaldehyde and Mowiol were from Electron Microscopy Science. Glass microscope slides and NH_4Cl were from Carlo Erba. The anti-human CD10, CD19 and CD20 monoclonal antibodies were form BioLegend. The anti-CD38 and anti-GAPDH monoclonal antibodies were from Pierce and AbD Serotec, respectively. The Alexa 546-conjugated anti-mouse goat antibody, and α-MEM and RPMI media were from Life Technologies. Hoechst 33342 was from Invitrogen. Nitrocellulose was from Millipore, and the ECL reagents were from Amersham.

Isolation of B lymphocyte populations from peripheral blood of healthy volunteers or leukemia patients. Normal B-cell-enriched fraction of lymphocytes was obtained using EasySep Negative selection kits (Stemcell Technologies Inc) for heparinized venous blood obtained from healthy volunteers, as previously described [65], and according to the manufacturer instructions. B-ALL cells were collected from the peripheral blood of three patients. After the samples were withdrawn, mononuclear cells were purified using Ficoll-Hystopaque 1.077 g/mL density gradient centrifugation. As the B-ALL cells were then around 95% of the purified fraction, no further step was used for these B-ALL cells. Before the RS, the cells were treated with 10 mg/mL DNAse-I solution (Stemcell Technologies Inc) to digest the DNA from damaged cells. This step was important to reduce viscosity,

and to remove membrane-bound DNA fragments that could interfere with the RS analysis. Informed consent for the use of biological samples for research purposes was obtained from both the healthy volunteers and the patients with B-ALL. The lymphocytes in the B-enriched fractions were routinely >98% surface immunoglobulin positive, as assessed by flow cytometry (see Supplementary Fig. 1).

Immunofluorescence microscopy sample preparation. RS4;11 (6 $\times 10^4$), REH (1 $\times 10^5$), and MN60 (3 $\times 10^4$) cells were suspended in 1 mL culture medium, plated onto 15-mm coverslips in a 24-well plates for 24 h, and then fixed with 2% paraformaldehyde (supplemented with 2 µg/mL Hoechst 33342, as the DNA dye) overnight at 4 °C. The cells were washed three times with phosphate-buffered saline (PBS), incubated in blocking solution (0.5% BSA, 0.05% saponin, 50 mM NH_4Cl, in PBS, pH 7.4) for 20 min at room temperature, and then incubated with the specified antibodies (CD38 1:50; CD20, CD19 1:10) diluted in blocking solution, overnight at 4 °C. After three washes with PBS, the cells were incubated with an Alexa 546-conjugated anti-mouse goat antibody (1:400 diluted in blocking solution) for 45 min at room temperature, and then washed again three times with PBS and twice with sterile water, to remove salt. The coverslips were mounted on glass microscope slides with Mowiol. Confocal images were acquired.

Western blotting. RS4;11 (6 $\times 10^4$), REH (1 $x10^5$), and MN60 (3 $\times 10^4$) cells were suspended in 2 mL cultured medium in 6-well plates for 72 h and then pelleted, washed tree times with PBS and lysed with 100 µL Laemmli buffer. The total proteins were separated on 10% SDS-PAGE, transferred onto nitrocellulose, and subjected to Western blotting. The nitrocellulose filters containing the proteins of interest were incubated in TTBS buffer [0.05% (w/v) Tween 20, 150 mM NaCl, 20 mM Tris-HCl, pH 7.5, supplemented with 1% (w/v) BSA] for 1 h at room temperature, and then with the primary antibody (CD38, 1:1000; CD10, 1:250; CD19, 1:500 in the same buffer) for overnight incubations at 4 °C. The primary antibodies were then removed and the strips were washed twice in TTBS, for 10 min each. The strips were incubated for 1 h at room temperature with the HRP-conjugated anti-mouse secondary antibody (1:5,000 in TTBS buffer), washed twice in TTB for 10 min each, and twice in TBS buffer (150 mM NaCl, 20 mM Tris-HCl pH 7.5). After washing, the strips were processed for ECL-based detection, according to the ECL reagents manufacturer instructions.

Flow Cytometry analysis RS4;11, REH and MN60 cell lines, 2 $x10^5$ cells were diluted in 100 µL of DPBS buffer supplemented with 1% BSA and incubated at 4°C for 30 min with appropriate amount of monoclonal antibodies, following the manufacturer instructions (provided by Beckman-Coulter). The combination of antibodies used are: i) anti-CD20 Fluorescein isothiocyanate with anti-CD19 Phycoerythrin; and ii) anti-CD10 Phycoerythrin with anti CD38 Phycoerythrin;-Cy7. All antibodies for flow cytometry analysis were provided from Beckman-Coulter. After incubation with these monoclonal antibodies, the cells were washed in DPBS with 1% BSA and diluted in 500 µL of the same solution for flow cytometry acquisition. At least 20.000 events were recorded for each experimental point on a FC-500 flow cytometer (Beckman-Coulter). Cells stained with irrelevant monoclonal immunoglobulin reagents conjugated with the same fluorochromes were used as negative controls.
